# Special Issue “Nuclear Physics: Effects of Radiation on Materials”

**DOI:** 10.3390/ma15217498

**Published:** 2022-10-26

**Authors:** Alberto Aloisio, Marcello Campajola

**Affiliations:** 1 Department of Physics “E. Pancini”, University of Naples “Federico II”, 80138 Napoli, Italy; 2 Istituto Nazionale di Fisica Nucleare (INFN), Sezione di Napoli, 80126 Napoli, Italy; 3 CNR-SPIN, 80078 Pozzuoli, Italy; 4 Task Force di Bioelettronica, University of Naples “Federico II”, 80138 Napoli, Italy

Understanding radiation damage in materials has been a topic of great interest for many years and in multiple scientific sectors. Radiation, in the form of charged particles, neutrons, or electromagnetic radiation, is known to accelerate the aging of materials and to degrade their mechanical, electrical, and optical properties. Several fields of application may suffer from an unfavorable radiation environment that can affect the performance of the employed components, inducing malfunctions or, ultimately, functional failure. Understanding radiation damage mechanisms and searching for more radiation-resistant materials is, therefore, paramount to guarantee lasting operation in harsh radiation environments. Depending on the specific application, radiation environments may differ by the complexity of the radiation fields, fluences, and energy distributions. Several applications foresee operations at extremely high radiation doses. Notable examples are (a) high-energy physics experiments at accelerators or colliders; (b) spacecraft missions; (c) nuclear reactors; and (d) medical physics diagnosis/therapy applications. Among these, radiation is considered particularly harmful for materials employed as structural and functional components, such as plastic, glass, oil, grease, semiconductor material devices, as well as living tissues.

The research field on radiation damage to materials can rely on a large and multidisciplinary scientific community that releases a wealth of public results and data. Although a large number of studies has already been published, upcoming increasingly challenging applications and the continuous scientific and technological progress in developing new materials and novel devices require up-to-date studies in line with the times. This became particularly clear, as an example, in the challenge of choosing and developing components and detectors to be used in ongoing upgrades of existing high-energy physics experiments at the Large Hadron Collider (LHC) and future particle colliders. A further case that is urgently calling for new studies is related to recent achievements in novel device developments, such as optoelectronic film materials devices, for which little is known about their response to radiation exposure.

In this respect, this Special Issue aims at collecting state-of-the-art developments in all such topics, addressing the modeling and experimental analysis of micro- and nanostructural material changes, the impact of radiation on the macroscopic behavior of complex devices and living tissues, as well as the design and performance of innovative sensors and radiation detectors.

